# Chemical Composition and Evaluation of the Biological Properties of the Essential Oil of the Dietary Phytochemical *Lippia citriodora*

**DOI:** 10.3390/molecules23010123

**Published:** 2018-01-12

**Authors:** Eleni Fitsiou, Gregoria Mitropoulou, Katerina Spyridopoulou, Manolis Vamvakias, Haido Bardouki, Alex Galanis, Katerina Chlichlia, Yiannis Kourkoutas, Mihalis Ι. Panayiotidis, Aglaia Pappa

**Affiliations:** 1 Department of Molecular Biology and Genetics, Democritus University of Thrace, University Campus, Dragana, 68100 Alexandroupolis, Greece; elenfits@gmail.com (E.F.); grigoriamitropoulou@gmail.com (G.M.); aikspiridopoulou@gmail.com (K.S.); agalanis@mbg.duth.gr (A.G.); achlichl@mbg.duth.gr (K.C.); ikourkou@mbg.duth.gr (Y.K.); 2 VIORYL S.A., Chemical & Agricultural Industry, Research S.A., 19014 Afidnes, Greece; vamvakias@vioryl.gr (M.V.); bardouki@vioryl.gr (H.B.); 3 Department of Applied Sciences, Northumbria University, Newcastle Upon Tyne NE1 8ST, UK

**Keywords:** *Lippia citriodora*, citral, composition, antimicrobial, antioxidant, antiproliferative

## Abstract

The aim of the study was to characterize the chemical composition and biological properties of the essential oil from the plant *Lippia citriodora* grown in Greece. The essential oil volatiles were analyzed by gas chromatography–mass spectrometry GC-MS indicating citral as the major component. Τhe antimicrobial properties were assayed using the disk diffusion method and the minimum inhibitory and non-inhibitory concentration values were determined. *Listeria monocytogenes*, *Staphylococcus epidermidis*, *Staphylococcus aureus*, *Saccharomyces cerevisiae*, and *Aspergillus niger* were sensitive to *Lippia citriodora* oil, but not *Escherichia coli*, *Salmonella* Enteritidis, *Salmonella typhimurium*, and *Pseudomonas fragi*. Adversely, all microbes tested were sensitive to citral. 2,2-Diphenyl-1-picrylhydrazyl (DPPH) and 2,2′-azino-bis(3-ethylbenzothiazoline-6-sulphonic acid) (ABTS) assays were used to assess direct antioxidant activity, which proved to be weak for both agents, while comet assay was utilized to study the cytoprotective effects against H_2_O_2_-induced oxidative damage in Jurkat cells. Interestingly, the oil showed a more profound cytoprotective effect compared to citral. The antiproliferative activity was evaluated in a panel of cancer cell lines using the sulforhodamine B (SRB) and 2,3-bis(2-methoxy-4-nitro-5-sulfophenyl)-*S*-(phenylamino) carbonyl-2-tetrazolium hydroxide (XTT) assays and both agents demonstrated potent antiproliferative activity with citral being more cytotoxic than the oil. Taken together, the essential oil of *Lippia citriodora* and its major component, citral, exert diverse biological properties worthy of further investigation.

## 1. Introduction

Nowadays, public interest for natural products has increased; thus research has focused on exploring their activities as therapeutic agents for a broad range of pathological conditions including various types of cancer. Phytochemicals have been shown to reduce cancer cell viability and migration and interfere with intracellular pathways by altering the expression profiles of many genes [1,2,3]. Based on the promising biological properties that many compounds possess and their few side-effects, dietary natural products have attained a significant interest in being used as protective and therapeutic agents against cancer. In addition, there is also a growing trend in discovering new compounds of natural origin as food preservatives with many of them possessing such potential [4]. Thus, there is an ever-increasing trend in identifying novel natural compounds with biological significance for their exploitation both in the pharmaceutical and food industries.

*Lippia citriodora*, commonly known as lemon verbena, belongs to the *Lippia* genus, which contains around 200 species. It was originally cultivated in South and Central America and was brought to Europe in the 17th century [5]. There are published data on the activities of different extracts of the plant prepared by infusion or decoction [6,7,8]. Its leaves are mainly used for the preparation of infusions which have been utilized for the relief of gastrointestinal symptoms. Ιn addition, antispasmodic, diuretic, and sedative properties have also been described; however, the literature is sparse regarding the biological activities of the essential oil extracted from the plant. 

Therefore, the aim of this study was to identify the composition of the essential oil of *Lippia citriodora* isolated from Greek plants and characterize its biological activities. We report here the (i) antimicrobial; (ii) antioxidant; (iii) cytoprotective (against H_2_O_2_-induced oxidative damage), and (iv) antiproliferative properties associated with the essential oil fraction derived from the leaves and stems of the plant. Finally, its major component, citral, was also evaluated for its biological properties in vitro and compared to the essential oil. To our knowledge, this is the first detailed study on distinct biological properties of the essential oil fraction of *Lippia citriodora*.

## 2. Results and Discussion

### 2.1. Chemical Composition

A total of 43 compounds, representing 87% of the total chromatographic area, were identified (Table 1). Neral (*cis*-citral) and geranial (*trans*-citral) reported as citral (which is the sum of the two isomers) accounted for 17.2% and 26.4%, respectively. Other major compounds identified were nerol (8.0%), geraniol (5.7%), spathulenol (3.3%), 1.8 cineol (3.2%) and limonene (2.2%). The results of the present study are in accordance with existing literature as geranial is reported as one of the main components of *Citriodora* species essential oil [9].

### 2.2. Antimicrobial Activitys

The antimicrobial activity of *Lippia citriodora* essential oil and its main constituent was evaluated against seven common food spoilage and pathogenic bacteria, as well as against *S. cerevisiae* and *A. niger*, which have been used previously as model systems in food spoilage.

Initially, the disk diffusion method was applied and subsequently the minimum inhibitory concentration (MIC) and non-inhibitory concentration (NIC) values were assessed using an established optical density method, which combines the absorbance measurements with the common dilution method. Non-linear regression analysis was used to fit the data using a previously-published model [10,11]. The data indicated that only *S. epidermidis*, *S. aureus*, and *L. monocytogenes* were sensitive to *Lippia citriodora* essential oil, although all bacteria were sensitive to citral (Table 2). Of note, large inhibition zones were observed in both *S. cerevisiae* uvaferm NEM (Table 2) and *A. niger* 19111 for both agents (inhibition zone of 20 ± 0.5 mm for 100 spores/plate initial inoculum) (the inhibition zones disappeared after one day οf incubation), which were similar to the positive control [12].

In accordance with the results of the disc diffusion method, MIC and NIC determination documented the effective growth inhibition of *Lippia citriodora* essential oil against *S. epidermidis*, *S. aureus*, and *L. monocytogenes* and citral against all bacteria tested (Table 3), although MIC and NIC values were significantly (*p* < 0.05) higher compared to ciproxin, which was used as positive control [12]. Noticeably, the oil was more effective compared to citral, as significantly (*p* < 0.05) lower MIC and NIC values were recorded. Similar results reporting high antimicrobial activity of *Lippia citriodora* essential oil and extracts were previously reported [6,13,14]. However, they were only limited to disc or well diffusion assays and no MIC and NIC values were determined. The antimicrobial activity of the essential oil could be attributed to the action of its main constituent, although possible antagonistic effects should not be excluded [15,16]. Such effects must be further studied using model systems.

### 2.3. Antioxidant Activity

In the present study, the DPPH and ABTS assays were used for the evaluation of the antioxidant capacity of the oil and citral. More particularly, increasing concentrations of the essential oil (0.0046–46 mg/mL) and citral (0.0045–45 mg/mL) were incubated with DPPH and ABTS for 30 and 15 min, respectively. The IC_50_ values for the essential oil were 6.3 ± 0.25 mg/mL for the DPPH assay and 3.08 ± 0.3 mg/mL using the ABTS method. Citral did not show any significant antioxidant activity (maximum DPPH inhibition 3.9% and ABTS inhibition 22.45%; Table 4).

The radical scavenging activity of citral has been tested before using the DPPH and ABTS assays, showing significant activity (IC_50_ values ranging from 30 to 260 μg/mL), in disagreement with our results [17,18,19]. Compared to lemon verbena oil, its activity was weaker showing that the oil probably owes its radical scavenging activity to its other constituents, as this is the case for other oils as well [20]. Both assays showed the same trend, however, in the ABTS method, both agents demonstrated a more potent antioxidant potential. Regardless of the assays used, their activity was shown to be less when compared to the potent antioxidant, ascorbic acid (Table 4). Differences in the results from the two assays have been reported before, where extracts were found to be more effective as ABTS rather than DPPH scavengers [21,22]. These differences can be attributed to a variety of factors, such as stereoselectivity of the radicals, solubility of the extracts in the different systems, and underlying mechanism(s) of action of the reaction [23,24].

### 2.4. Genotoxic or Cytoprotective (Against H_2_O_2_-Induced Oxidative Damage) Activity

**Figure 1:**
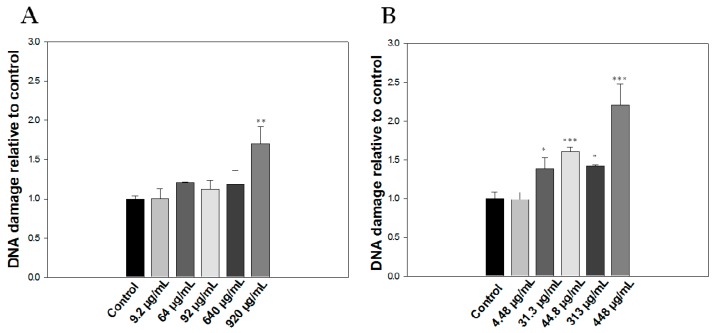
Detection of DNA damage caused by *Lippia citriodora* oil and citral in Jurkat cells using comet assay. Jurkat cells (2 × 10^4^) were incubated with *Lippia citriodora* oil (**A**) or citral (**B**) for 20 min at room temperature. Results are shown as Mean ± S.D. * *p* < 0.05, ** p < 0.01, *** p < 0.001.

The genotoxic effects of the oil fraction or citral were investigated using the comet assay. We observed that the oil fraction demonstrated a genotoxic effect only at the highest concentration used (920 μg/mL) in Jurkat cells after 20 min of incubation, which was approximately one and a half times the levels of DNA damage of the control sample (Figure 1A). Conversely, citral exhibited a non-concentration dependent genotoxicity that reached the levels of the maximum oil-induced DNA damage at a very low concentration (44.8 μg/mL) (Figure 1B). In addition, it caused a 2.2-fold DNA damage compared to the control group at the highest concentration used (448 μg/mL), which was half the maximum concentration of the oil (920 μg/mL).

**Figure 2:**
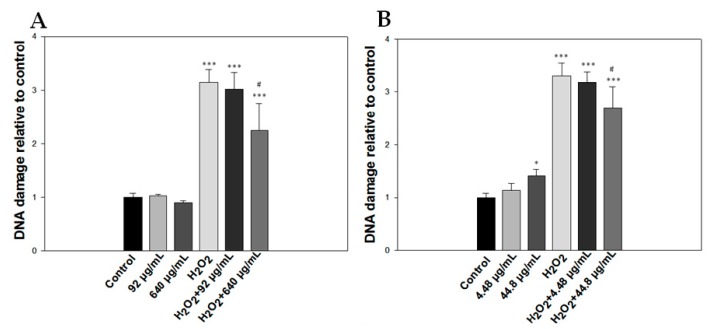
Detection of the protective effect of *Lippia citriodora* oil and citral on H_2_O_2_-treated Jurkat cells. Jurkat cells (2 × 10^4^) were preincubated with *Lippia citriodora* oil (**A**) or citral (**B**) for 20 min before treatment with H_2_O_2_ (6.66 μg/mL) for 20 min at room temperature. Results are shown as mean ± S.D. ^#^ *p* = 0.013 H_2_O_2_ vs. H_2_O_2_ and 640 μg/mL essential oil, ^#^ *p* < 0.05 relative to H_2_O_2_, *** *p* < 0.001 relative to control.

Moreover, we used two concentrations of both agents (essential oil, 92 and 640 μg/mL; citral, 4.48 and 44.8 μg/mL) to assess the protective effect of each agent against H_2_O_2_-induced oxidative damage. Overall, both agents exhibited similar levels of protection only at the highest concentration used, with the oil providing slightly more protection than citral (2.24-fold vs. 2.7-fold the DNA of control sample, respectively) (Figure 2A,B).

The oil from *Lippia citriodora* together with citral have been previously evaluated for their antigenotoxic effect against ultraviolet radiation-induced DNA damage using the SOS chromosome test, where they both showed antigenotoxicity [25]. Citral has shown diverse toxicity using different test systems. For instance, it not did exhibit direct pro-oxidant effect when testing oxygen uptake in erythrocytes exposed to *tert*-butyl hydroperoxide (t-BHP) [26], nor did it reduce the viability of rat small intestine epithelial cells after 24 h of incubation [17]. When using the *Salmonella* mutagenicity testing, Gomes-Carneiro et al., demonstrated that citral was toxic at concentrations higher than 600 μg/mL, whereas it did not demonstrate any such genotoxicity when utilizing the SOS chromotest [27,28]. Moreover, in a similar study, citral was shown to reduce the viability of human lymphocytes over 100 μg/mL using the MTT assay and cause statistically significant DNA damage at even lower concentrations (>25 μg/mL), in accordance with our results [29]. 

### 2.5. Antiproliferative Activity 

**Figure 3:**
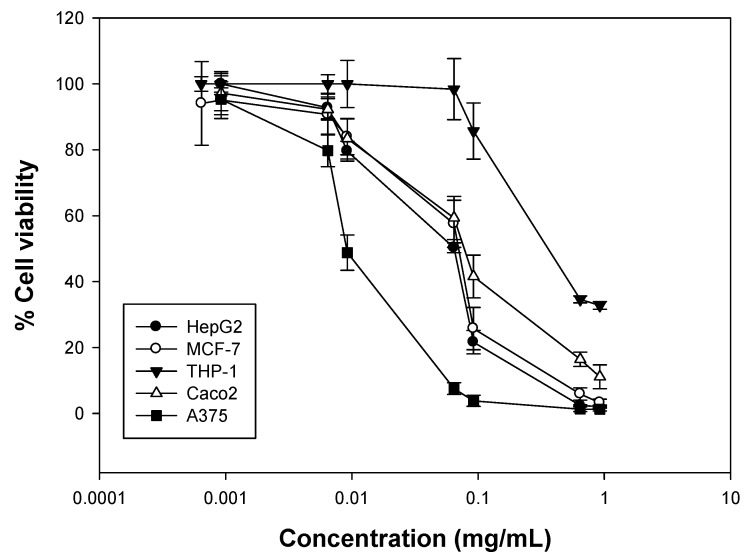
Antiproliferative activity of *Lippia citriodora* oil against a panel of five human cancer cell lines. Cancer cells were incubated with increasing concentrations of citral for 72 h. Estimation of cell viability was determined by the SRB assay. Representative figures of at least three experiments.

Increasing concentrations of the essential oil fraction (0.64–920 μg/mL) or citral (0.63–900 μg/mL) were incubated with different human cancer cell lines (for 72 h) and SRB or XTT assays were employed to determine cell viability. Overall, it was observed that the oil fraction was most cytotoxic against the A375 (melanoma) cells (EC_50_ = 9.1 ± 0.6 μg/mL), an activity eight to ten times higher compared to the other cell lines tested. In addition, it showed similar viability levels against HepG2 (hepatocellular carcinoma), MCF-7 (breast adenocarcinoma) and Caco2 (colon adenocarcinoma) cells (EC_50_ = 74 ± 2.8 μg/mL, 89 ± 1.4 μg/mL and 71 ± 2.6 mg/mL, respectively), while it was slightly less cytotoxic against THP-1 (leukemic monocytes) cells (EC_50_ = 111 ± 3.6 μg/mL) (Figure 3 and Table 5). Finally, the oil fraction demonstrated modest cytotoxicity to the lines tested compared with etoposide, a known chemotherapeutic agent. To our knowledge, there are no published data on the cytotoxicity of the essential oil of *Lippia citriodora* against HepG2 and Caco2 cell lines. In a study in 2010, Escobar et al. showed the effect of *Lippia citriodora* oil (from Colombian plants) against Vero and THP-1 cells where the EC_50_ value for THP-1 was >100 μg/mL, in accordance with our results [30]. There is also one study describing the cytotoxic activity of *Aloysia citriodora* oils from different regions of Morocco against MCF-7 cells after a 48 h incubation, where the EC_50_ values ranged between 35 and 70 μg/mL, whereas there was no cytotoxicity observed against PBMCs [31]. In this case, the oils demonstrated higher activity compared to our oil, which may be attributed to the differences in their composition, as the Moroccan oils had *β*-spathulenol, *trans*-caryophyllene oxide and ar-curcumene as major components.

**Figure 4:**
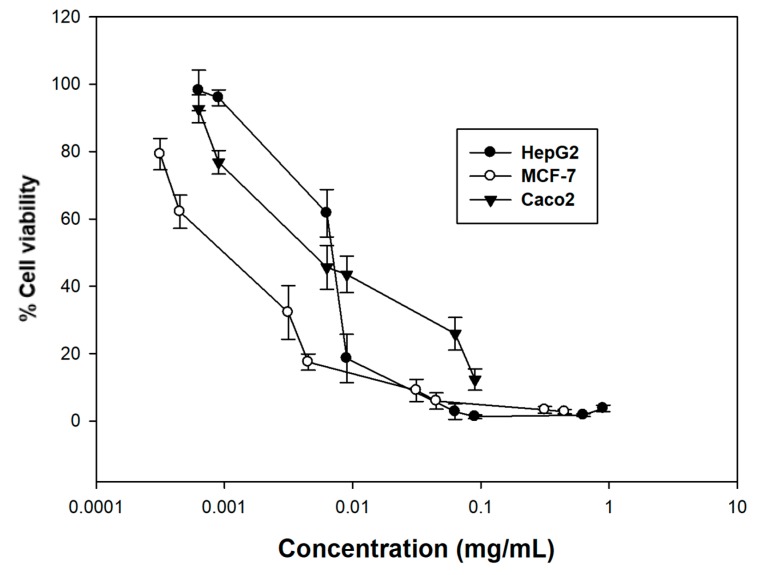
Antiproliferative activity of citral against a panel of three human cancer cell lines. Cancer cells were incubated with increasing concentrations of citral for 72 h. Estimation of cell viability was determined by the SRB assay. Representative figures of at least three experiments.

On the other hand, citral was significantly more potent against all cell lines tested, an observation which could owe to an antagonistic effect between the components of the essential oil. In general, MCF-7 cells were the most sensitive ones when subjected to citral (EC_50_ = 1.3 ± 0.19 μg/mL), followed by Caco2 and HepG2 cells (EC_50_ = 3.7 ± 0.21 μg/mL and 7 ± 0.35 μg/mL, respectively) (Figure 4 and Table 5). To this end, another study utilizing citral (obtained commercially) and also tested against MCF-7 cells showed an EC_50_ value of 22 μg/mL [32], an effect significantly smaller than that of our study, which may be attributed to the different cell viability assay used. In another study, citral has been tested against HepG2 cells after 24 and 48 h of incubation (EC_50_ = 30.129 and 14.67 μg/mL, respectively), in accordance with our results, where after 72 h the EC_50_ value was even lower, suggesting a time-dependent effect [19], while it has also been tested for its cytotoxicity against a range of human cancer cell lines including breast carcinoma, glioblastoma, malignant melanoma, and colon carcinoma after a 72 h incubation, exhibiting potent activity [18].

## 3. Materials and Methods

### 3.1. Plant Material

Plant material was purchased by Vioryl S.A. from a local area herbal market (Afidnes, Athens, Greece) and the species was confirmed by a professional botanist. Plants were small shrubs of almost 60 cm height. They were kept in pots until the first inflorescence appeared and leaves and stems were collected.

### 3.2. Chemicals and Reagents

Brain heart infusion (BHI) broth, malt extract agar, and Ringer’s solution were obtained from LABM (Heywood, UK). Ciproxin was obtained from Oxoid Ltd. (Basingstoke, UK) and amphotericin B from Mast Group Ltd. (Merseyside, UK). Dulbecco’s Modified Eagle’s Medium (DMEM), DMEM high glucose, RPMI media, and low melting agarose were purchased from Gibco^®^ (Gaithersburg, MD, USA). Fetal bovine serum (FBS), trypsin, penicillin/streptomycin, trypan blue 0.5%, and phosphate-buffered saline (PBS) were purchased from Biosera (Boussens, France). Dimethyl sulfoxide (DMSO) and propidium iodide were purchased from Biotium (Hayward, CA, USA), while hydrogen peroxide, ABTS, potassium persulfate, ascorbic acid, sulforhodamine B (SRB), Trizma base, and etoposide were purchased from Sigma-Aldrich (St. Louis, MO, USA). Trichloroacetic acid (TCA) was obtained from MP Biomedicals (Santa Ana, CA, USA). Acetic acid and ethanol were purchased from Scharlau (Barcelona, Spain) and DPPH from Calbiochem^®^ (Darmstadt, Germany).

### 3.3. Essential Oil Extraction and GC/MS Analysis

The essential oil was obtained by hydrodistillation at VIORYL S.A. facilities (Afidnes, Athens, Greece) directly after the harvesting period, taking into account the seasonality of the plant. All plants were harvested during May and June, and no further drying process was used. Chopped leaves and stems were collected by hand, followed by hydrodistillation with a Dean Stark apparatus. Plant material was covered with 6 L of distilled water, while the extraction process took place for 8 h at a temperature of 90–120 °C. Isolated essential oil was dried with Na_2_SO_4_ and sealed in vials for further use. Analysis was carried out with a GC-MS (GC: 6890A, Agilent Technologies, Santa Clara, CA, USA; MSD: 5973, Agilent Technologies, Santa Clara, CA, USA) using a Factor Four VF 1 ms column (25 m, 0.2 mm i.d., 0.33 μm film thickness, Agilent Technologies, Santa Clara, CA, USA). A volume of 0.1 μL of essential oil was directly injected and a 1:100 split ratio was applied. Oven temperature was set at 50 °C for 1 min, followed by a temperature gradient of 2.5 °C/min to 160 °C (for 20 min), then raised to 250 °C at 50 °C/min with a final isothermal period of 15 min. Helium was used as the carrier gas (flow rate 1 mL/min). Injector and transfer line temperatures were set to 200 °C and 250 °C, respectively. The mass spectrometer operated in the electron impact mode with the electron energy set to 70 eV. Identification of the compounds was carried out according to the standard method of Kováts Indices and comparison of volatiles mass spectra to Willey/NIST 0.5 and in-house created libraries (VIORYL S.A.).

### 3.4. Microbial Strains

*Salmonella enterica* subsp. *enterica* ser. Enteritidis FMCC Β56 PT4 (kindly provided by Prof. Nychas G.J.E., Agricultural University of Athens, Athens, Greece), *Salmonella enterica* subsp. *enterica ser. typhimurium* DSMZ 554, *Listeria monocytogenes* NCTC 10527 serotype 4b, *Escherichia coli* ATCC 25922, *Staphylocοccus epidermidis* FMCC B-202 C5M6 (kindly provided by Dr. Nisiotou A., Athens Wine Institute, ELGO-DIMITRA, Athens, Greece) and *Staphylococcus aureus* ATCC 25923 were grown in BHI at 37 °C for 24 h. Likewise, *Pseudomonas fragi* 211 (kindly provided by Prof. Nychas G.J.E., Agricultural University of Athens, Greece) was grown in BHI broth at 25 °C for 24 h. *Saccharomyces cerevisiae* uvaferm NEM (Lallemand, Montreal, QC, Canada) was grown in YPD broth (yeast extract, 10 g/L; glucose, 20 g/L; and peptone, 20 g/L) at 28 °C for three days. *Aspergillus niger* 19111 (kindly provided by Prof. Nychas G.J.E., Agricultural University of Athens) was grown on malt extract agar for seven days at 37 °C. 

### 3.5. Antimicrobial Assays

The antimicrobial activity of the tested essential oil and determination of minimum inhibitory concentration (MIC) and non-inhibitory concentration (NIC) based on the Lambert-Pearson model (LPM) [10,11] were monitored using previously published methodologies [12,33]. In brief, the effect on the growth, measured by the optical density method, is manifested by a reduction in the area under the OD/time relative to a control well at any specified time (supplementary material). By calculating the area using the trapezoidal rule (Equation (1)), the relative amount of growth were obtained using the ratio of the test area to that of the control, termed the fractional area, *fa*. Data were fitted to the LPM using non-linear least squares regression analysis assuming equal variance.

(1) $$fa=\exp\left[-{\left(\frac{x}{P_{1}}\right)}^{P_{2}}\right]$$

where *fa* is the fractional area, *x* is the inhibitor concentration (μg/mL), *P*_1_ is the concentration at maximum slope (of a logx vs*. fa* plot), and *P*_2_ is a slope parameter.

*MIC* was defined as the intercept of the concentration axis to the tangent at the maximum gradient of the *fa*/log concentration curve (Equation (2)):

(2) $$MIC=P_{1}\exp\left(\frac{1}{P_{2}}\right)$$

*NIC* was defined as the intercept of the tangent at the maximum gradient of the *fa*/log concentration curve to the *fa* = 1 contour (Equation (3)):

(3) $$NIC=P_{1}\exp\left(\frac{1-e}{P_{2}}\right)$$

### 3.6. Antioxidant Activity

#### 3.6.1. DPPH Assay

The radical scavenging activity of the essential oil and citral was estimated using the free radical DPPH, as previously described [12]. Increasing concentrations of the essential oil (0.0046–46 mg/mL) and citral (0.0045–45 mg/mL) were prepared using DMSO as the solvent. Absorbance was measured at 517 nm using an ELISA plate reader (EnSpire Multimode Plate Reader, Perkin Elmer, Waltham, MA, USA). All determinations were performed in triplicates. The % inhibition of the DPPH radical for each concentration was determined by making use of the following formula: % DPPH radical scavenging activity = [(OD_control_ − OD_sample_)/OD_control_)] × 100. 

#### 3.6.2. ABTS Assay

The ABTS de-coloration assay was performed as previously described [12,34]. The % inhibition of the ABTS radical for each concentration is expressed in two ways; First, by making use of the following formula: % ABTS radical scavenging activity = [(OD_control_ − OD_sample_)/OD_control_)] × 100. Next, a standard curve based on the percentage of ABTS radical scavenging activity of known concentrations of ascorbic acid expressed in μM was prepared and the concentrations of the samples were calculated using linear regression analysis and the results were also expressed as micromoles ascorbic acid equivalent per gram of essential oil (μmolesEA/g), by making use of the following formula: C = (cxD)/Ci. C, concentration of antioxidant compounds in μmolesEΑ/g; c, concentration of sample read (in micromoles per liter); D, dilution factor; Ci, concentration of stock solution (in grams per liter).

### 3.7. Cell Lines and Cell Cultures

The human cancer cell lines Caco2 (colorectal adenocarcinoma), HepG2 (hepatocellular carcinoma), MCF-7 (breast adenocarcinoma), ΤΗP-1 (leukemic monocytes), Jurkat (acute T cell leukemia), and A375 (malignant melanoma) were obtained from the American type culture collection (Rockville, MD, USA). HepG2 and MCF-7 cells were grown and maintained in DMEM, A375 cells in DMEM high glucose (4500 mg/L), whereas the medium RMPI was used for the Caco2, Jurkat, and THP-1 cell lines. All media were supplemented with 10% FBS, penicillin (100 U/mL), and streptomycin (100 μg/mL) and were incubated at 37 °C in a humidified atmosphere of 95% O_2_ and 5% CO_2_. Stock cultures were passaged at 2- to 3-day intervals. Cells were seeded at a density of 5.0 × 10^3^ cells per well in 96-well plates for the SRB assay. THP-1 cells were seeded at a density of 2.0 × 10^3^ cells per well in round bottom 96-well plates for the XTT assay.

### 3.8. Single Cell Gel Electrophoresis Assay (Comet Assay)

The alkaline version of the single-cell gel electrophoresis assay was used to evaluate DNA damage of the essential oil and citral, as well as their protective effect from H_2_O_2_-induced oxidative damage. Briefly, Jurkat cells (2 × 10^4^ cells/sample in PBS) were maintained on ice. Cells were treated with increasing concentrations of the essential oil or citral alone for 20 min or followed by treatment with H_2_O_2_ (6.66 μg/mL) for another 20 min at room temperature. Comet assay was performed as previously described [35]. The slides were processed for evaluation on a Zeiss Axio Scope.A1 fluorescence microscope (Oberkochen, Germany). The overall DNA damage was calculated in arbitrary units. Results were expressed as % DNA damage relative to control.

### 3.9. Cell Viability Assays

#### 3.9.1. SRB Assay

The viability of the human cancer cells HepG2, Caco2, MCF-7, and A375 after treatment with the essential oil and its major component was determined using the SRB assay as previously described [12]. Cells were plated in 96-well plates and treated with increasing concentrations of the oil (0.64–920 μg/mL) and citral (0.63–900 μg/mL) (dissolved in DMSO, 1:1 *v*/*v*) for 72 h. 

#### 3.9.2. XTT Assay 

The viability of THP-1 cells was determined by the XTT assay as previously described [12,36]. Cells were seeded in a 96-well-plate and following an overnight incubation they were treated with increasing concentrations of the oil (0.64–920 μg/mL) or citral (0.63–900 μg/mL) (dissolved in DMSO, 1:1 *v*/*v*) for 72 h. At the end of the incubation, the XTT solution was added, and plates were incubated further for 4 h before reading the absorbance at 450 nm by a microplate reader (EnSpire Multimode Plate Reader, Perkin Elmer, Waltham, MA, USA). 

### 3.10. Data Analysis

All experiments were performed at least in triplicate. For MIC and NIC determination, each experiment was performed at least 4 times, and standard deviation was calculated by Fig.P software (Fig.P Software Incorporated, Hamilton, ON, Canada). Significance was established at *p* < 0.05 and the results were analyzed for statistical significance with analysis of variance (ANOVA). Duncan’s multiple range test was used to determine significant differences among results using Statistica v.10.0. The IC_50_ (inhibition concentration) and EC_50_ (efficient concentration) values were calculated as previously described [12]. For comet assay, statistical differences between groups were evaluated by ANOVA followed by Dunnett’s or Tukey’s test. A level of *p* < 0.05 was considered statistically significant. All statistical analyses were performed using GraphPad Prism 5 (GraphPad Software, San Diego, CA, USA).

## 4. Conclusions

Citral is the major component of the essential oil of *Lippia citriodora* (obtained from Greek plants) and exhibited significant antimicrobial activity against all microbes tested in contrast to the oil fraction which was inactive against the gram negative bacteria. Our results also suggest that both the oil fraction and citral exhibit potent antiproliferative activities in vitro. More specifically, citral was more cytotoxic against all cancer cell lines utilized in the scope of this study while the oil fraction exhibited lower genotoxicity. Although both the oil and citral proved weak direct antioxidants as assessed by biochemical in vitro assays, they, nevertheless, exhibited antioxidant capacity in a cellular system demonstrated as a significant reduction of the H_2_O_2_-induced oxidative damage. Many of these properties are reported here for the first time, thus setting the basis for further investigations regarding the active components of the oil and the molecular mechanisms(s) underlying their mode of action. This is of utmost importance as the demand in identifying natural products with well-described biological properties for potential neutraceutical and pharmaceutical applications is constantly increasing.

## Figures and Tables

**Table 1:** Volatiles identified in the essential oil of *Lippia citriodora* and their relative percent (%) area.

KRI*	Compounds	% Area
795	*trans-*hex-2-enal	0.024
805	*cis-*hex-3-enol	0.084
819	*trans*-hex-2-enol	0.013
920	α-pinene	0.041
946	oct-1-en-3-one	0.072
954	6-methyl-hept-5-en-2-noe	2.278
956	oct-1-en-3-ol	1.434
971	octan-3-ol	0.079
972	myrcene	0.100
978	*cis*-hex-3-enyl acetate	0.071
1008	1,8-cineol	3.150
1010	limonene	2.166
1019	*cis*-b-ocimene	trace
1030	*trans*-b-ocimene	0.386
1043	sabinenehydrate	0.267
1077	nonanal	0.053
1080	linalol	0.396
1137	*cis*-isocitral	0.485
1165	*a*-terpineol	1.119
1212	nerol	8.047
1215	*cis*-citral	17.160
1219	piperitone	0.193
1241	geraniol	5.720
1246	*trans*-citral	26.404
1278	thymol or carvacrol	0.462
1324	eugenol	0.190
1340	geranic acid	0.195
1360	geranyl acetate	0.999
1366	*a*-copaene	0.263
1367	methyl eugenol	0.129
1373	*b*-bourbonene	0.199
1400	*a*-cedrene	0.283
1405	caryophyllene	1.439
1462	*d*-germacrene	1.150
1464	ar-curcumene	2.098
1479	zingiberene	0.536
1479	bicyclogermacrene	1.750
1504	cubenol A	0.215
1543	nerolidol	0.753
1551	spathulenol	3.279
1554	caryophyllene oxide	1.375
1607	*iso*-spathulenol	0.452
1611	T-cadinol	0.558

KRI*: Kovats Retention Indices.

**Table 2:** Antimicrobial activity of the *Lippia citriodora* essential oil against common food spoilage and pathogenic microbes monitored by the disk diffusion assay.

	*Lippia citriodora* Essential Oil		Citral	
	Initial Inoculum			
Microbial Species	5 log cfu/mL	7 log cfu/mL	5 log cfu/mL	7 log cfu/mL
*Salmonella* Enteritidis	0	0	10 ± 0.5	7 ± 0.3
*Salmonella typhimurium*	0	0	10 ± 0.3	8 ± 0.5
*Escherichia coli*	0	0	11 ± 0.7	7 ± 0.5
*Listeria monocytogenes*	12 ± 0.7	10 ± 0.3	20 ± 0.3	15 ± 0.5
*Staphylococcus epidermidis*	20 ± 0.25	16 ± 0.3	25 ± 0.5	19 ± 0.3
*Staphylococcus aureus*	13 ± 0.5	11 ± 0.7	23 ± 0.5	19 ± 0.3
*Pseudomonas fragi*	0	0	10 ± 0.5	7 ± 0.3
*Saccharomyces cerevisiae*	20 ± 0.5	12 ± 0.7	25 ± 0.7	18 ± 0.3

The inhibition zones were measured in mm.

**Table 3:** MIC and NIC (μg/mL) of *Lippia citriodora* essential oil and citral against common food spoilage and pathogenic bacteria. Ciproxin was used as control.

	*Lippia Citriodora* Essential Oil		Citral *		Ciproxin (Data Reproduced by Fitsiou et al. [12])	
Microbial species	MIC	NIC	MIC	NIC	MIC	NIC
*Salmonella* Enteritidis	-	-	7051 ± 26	6393 ± 18	0.976 ± 0.001	0.957 ± 0.001
*Salmonella typhimurium*	-	-	7603 ± 26	6121 ± 9	0.979 ± 0.001	0.964 ± 0.001
*Escherichia coli*	-	-	7024 ± 9	6340 ± 18	0.984 ± 0.001	0.956 ± 0.002
*Listeria monocytogenes*	1794 ± 9	179 ± 9	6919 ± 18	4981 ± 18	0.979 ± 0.001	0.968 ± 0.001
*Staphylococcus epidermidis*	1758 ± 11	538 ± 19	6954 ± 18	5779 ± 9	0.979 ± 0.002	0.957 ± 0.002
*Staphylococcus aureus*	923 ± 19	98 ± 9	6901 ± 18	4972 ± 9	0.982 ± 0.002	0.963 ± 0.003
*Pseudomonas fragi*	-	-	7112 ± 27	5235 ± 9	0.955 ± 0.001	0.940 ± 0.002

* Mixture of 40% *cis*- and 60% *trans*-citral.

**Table 4:** Antioxidant activity of the essential oil of *Lippia citriodora* and citral using the DPPH and ABTS assays.

	DPPH	ABTS	
	IC_50_ (mg/mL)	IC_50_ (mg/mL)	(μmolesEΑ/g) *
*Lippia citriodora* oil	6.3 ± 0.25	3.08 ± 0.3	3115.2
Citral	n.d.	n.d.	773.7
Ascorbic acid	0.0054 ± 0.00035	0.0054 ± 0.00041	-

Data are presented as Mean ± SD of at least three independent experiments, * micromoles ascorbic acid equivalent per gram of essential oil. Ascorbic acid was used as a positive control. n.d. = not determined.

**Table 5:** EC_50_ values of the essential oil of *Lippia citriodora* and its major component, citral, against different human cell lines. Etoposide was used as a positive control.

EC_50_ (μg/mL)					
	HepG2	Caco2	MCF-7	THP-1	A375
*Lippia citriodora* oil	74 ± 2.8	71 ± 2.6	89 ± 1.4	111 ± 3.6	9.1 ± 0.6
Citral	7 ± 0.35	3.7 ± 0.21	1.3 ± 0.19	-	-
Etoposide	0.60 ± 0.06	7.3 ± 0.63	1.67 ± 0.41	0.45 ± 0.013	-

Data are presented as mean ± SD of at least three independent experiments.

## Supplementary Materials

Supplementary materials are available online.
